# MPSTAN: Metapopulation-Based Spatio–Temporal Attention Network for Epidemic Forecasting

**DOI:** 10.3390/e26040278

**Published:** 2024-03-25

**Authors:** Junkai Mao, Yuexing Han, Bing Wang

**Affiliations:** 1 School of Computer Engineering and Science, Shanghai University, Shanghai 200444, China; maojk@shu.edu.cn; 2 Key Laboratory of Silicate Cultural Relics Conservation (Shanghai University), Ministry of Education, Shanghai 200444, China; 3 Zhejiang Laboratory, Hangzhou 311100, China

**Keywords:** metapopulation epidemic, epidemic forecasting, spatio–temporal features, graph attention networks

## Abstract

Accurate epidemic forecasting plays a vital role for governments to develop effective prevention measures for suppressing epidemics. Most of the present spatio–temporal models cannot provide a general framework for stable and accurate forecasting of epidemics with diverse evolutionary trends. Incorporating epidemiological domain knowledge ranging from single-patch to multi-patch into neural networks is expected to improve forecasting accuracy. However, relying solely on single-patch knowledge neglects inter-patch interactions, while constructing multi-patch knowledge is challenging without population mobility data. To address the aforementioned problems, we propose a novel hybrid model called metapopulation-based spatio–temporal attention network (MPSTAN). This model aims to improve the accuracy of epidemic forecasting by incorporating multi-patch epidemiological knowledge into a spatio–temporal model and adaptively defining inter-patch interactions. Moreover, we incorporate inter-patch epidemiological knowledge into both model construction and the loss function to help the model learn epidemic transmission dynamics. Extensive experiments conducted on two representative datasets with different epidemiological evolution trends demonstrate that our proposed model outperforms the baselines and provides more accurate and stable short- and long-term forecasting. We confirm the effectiveness of domain knowledge in the learning model and investigate the impact of different ways of integrating domain knowledge on forecasting. We observe that using domain knowledge in both model construction and the loss function leads to more efficient forecasting, and selecting appropriate domain knowledge can improve accuracy further.

## 1. Introduction

In the past few years, COVID-19 has emerged as a significant threat to both human life and the global economy. Due to its highly contagious nature, millions of people have been infected, leading to enormous pressure on healthcare systems and social order [1]. Thus, it is imperative for governments and public health departments to devise effective epidemic prevention strategies, and accurate forecasting of the outbreak’s future evolution is a critical factor for preventing disease transmission, mitigating its impact on public health and the economy, and enhancing the quality and efficacy of medical services [2]. Accurate forecasting can provide early warnings to help relevant departments take necessary preventive measures before an outbreak, effectively allocate medical resources, and support public health policies and decisions, such as social distancing measures and vaccination strategies. With the rapid development of deep learning, it has made significant advancements in various fields such as computer vision and data mining [3,4]. Developing deep learning models for epidemic forecasting would provide more accurate forecasting and benefit the efficacy of interventions during epidemics.

Traditional epidemic forecasting models use compartmental models constructed from differential equations to simulate the potential transmission dynamics of epidemics at the patch level, such as the SIR model [5], SEIR model [6], and their variants [7,8]. Taking the SIR model as an example, it is used to estimate the fluctuations in the number of susceptible, infected, and recovered individuals within a single patch to understand the dynamics of the epidemic in a particular patch. Many traditional time-series methods can directly forecast the temporal dependency of epidemic outbreaks, such as ARIMA [9] and SVR [10]. In recent years, deep learning has been widely used in the field of time-series forecasting, and several excellent models have been proposed, including LSTM [11], GRU [12], transformer [13], and neural ODE [14]. These models are designed to effectively handle the unique properties of time-series data, such as temporal correlation, periodicity, etc.

However, the above methods only consider the temporal dependence of the data and ignore the spatial dependence, which may lead to insufficiently accurate forecasting results. The reason is that the epidemic evolution of a patch is not only influenced by its own factors, such as the scale of infection and medical resources, but also by external factors, such as the mobility of people from other patches [15]. Therefore, it is crucial to consider spatial dependence to improve the accuracy of epidemiological trend analysis and forecasting. The development of graph-based algorithms provides researchers with a powerful tool for taking epidemic forecasting as a spatio–temporal forecasting problem [16,17]. Various methods [18,19,20] have been proposed for epidemic spatio–temporal forecasting. In essence, these methods construct a graph to predict multi-patch epidemics. Each patch is represented as a node, and each patch’s historical data, such as the infected cases, recovered cases, hospitalizations, and ICU admissions, are used as node features. By modeling the temporal and spatial dependencies in epidemic data, these methods can capture potential spatio–temporal correlations to predict future trends in the epidemic spreading. With the benefit of spatio–temporal forecasting work in the traffic flow field, most of the spatio–temporal models can also be directly applied to epidemic forecasting, such as [21,22,23].

**Figure 1:**
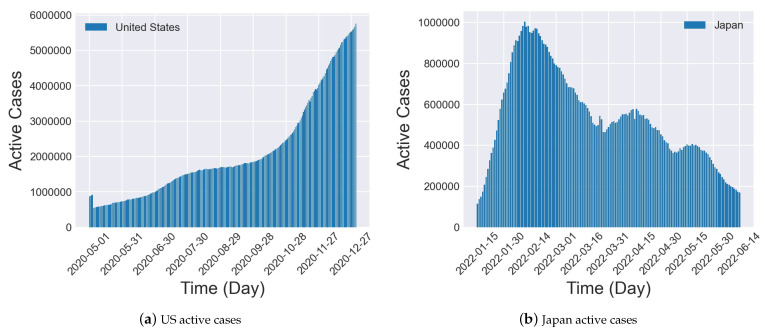
Illustration of active cases in the US and Japanese datasets.

Nevertheless, epidemiological evolutionary trends can vary considerably depending on the timing, region, and preventive measures of the epidemic outbreak. We show the number of active cases in the United States and Japan as recorded at different times in Figure 1. Both of these datasets are based on COVID-19 data. The US dataset is sourced from the Johns Hopkins University Coronavirus Resource Center [24] and covers the period from 1 May 2020 to 31 December 2020. The Japanese dataset is obtained from the Japan LIVE Dashboard [25] and spans the period from 15 January 2022 to 14 June 2022. As shown in Figure 1, these two datasets show completely different epidemiological evolutionary trends. Figure 1a indicates that the outbreak is ongoing, and Figure 1b indicates that the outbreak is under control, where the different trends reflect the vastly different transmission dynamics of the epidemic. Traditional spatio–temporal models only find a nonlinear mapping between input and output data and do not consider the underlying physical information, which also makes it difficult to provide stable and accurate forecasting in the face of complex trends [26]. In response to this issue, [27] points out that it is not reasonable to simply apply deep learning to epidemic forecasting. Furthermore, theory-guided data science demonstrates that incorporating domain knowledge into data-driven models helps improve algorithm performance [28]. Therefore, researchers have attempted to use epidemiological domain knowledge to help models better learn the underlying dynamics of epidemics. Some works, such as [29,30,31], incorporate single-patch epidemic models such as SIR and SIRD into spatio–temporal models, providing meaningful epidemiological context for neural networks and improving the performance of epidemic forecasting. However, they neglect inter-patch epidemic transmission, so some researchers [32] use population mobility data to construct a metapopulation epidemic transmission model and train the learning model using this domain knowledge.

Although existing methods have achieved success in this field, we find the following limitations:

(1) Most of the existing methods fail to make full use of the more reasonable epidemiological domain knowledge to help model training. They utilize domain knowledge that either ignores inter-patch interactions [29,30] or requires additional population mobility data to construct inter-patch interactions [32]. The latter approach relies heavily on population mobility data, but collecting population mobility data between patches is inherently challenging and inaccurate, which can also bias the model.
(2) Most of the existing domain-knowledge-based models do not analyze the effectiveness of domain knowledge on model training in detail. Most methods only apply epidemiological domain knowledge to the loss function [30,32], and some works apply epidemiological knowledge to model construction at the same time [31]. However, these methods do not analyze in detail the effectiveness of domain knowledge on model construction and the loss function separately for epidemic forecasting.

To address the above limitations, we propose a novel approach named metapopulation-based spatio–temporal attention network (MPSTAN). MPSTAN employs the MP-SIR model that considers inter-patch mobility to help spatio–temporal model training. Specifically, the MP-SIR physical model utilizes the neural network to learn physical model parameters both intra- and inter-patch, thus enabling adaptive construction of interactions between patches. Furthermore, we believe that different parameters are influenced by distinct types of information. The intra-patch parameters primarily represent the scale of the epidemic within a given patch, which reflects the temporal variations in population size for each state. The inter-patch parameters, on the other hand, capture the population mobility between patches and are also influenced by spatial information. Therefore, we design multiple parameter generators that include two fully connected layers. By separately passing embeddings containing temporal dependencies and spatio–temporal dependencies to these two respective fully connected layers, we adaptively learn the intra- and inter-patch parameters. In addition, we apply the physical model to model construction and the loss function of the MPSTAN model and thoroughly analyze the effectiveness of different ways of combining the physical model with the learning model for epidemic forecasting. Furthermore, a single physical model does not accurately represent the potential epidemiological dynamics in various real-world environments. For more accurate forecasting, selecting an appropriate epidemiological physical model tailored to the specific circumstances is necessary. In summary, the main contributions of this paper are as follows:

(1) We propose a metapopulation-based spatio–temporal attention network for epidemic forecasting. Specifically, we propose a metapopulation epidemic model with parameters adaptively learned through neural networks, which is then incorporated to guide neural network training. This spatio–temporal model does not rely on population mobility data, enabling it to accurately predict epidemic transmission.
(2) We design multiple parameter generators to learn the physical model parameters for the intra- and inter-patches separately. Due to the fact that different parameters represent different information, we utilize embedding representations containing diverse information to feed into each parameter generator separately in order to learn the corresponding physical model parameters.
(3) We reveal the significance of epidemiological domain knowledge in spatio–temporal epidemic forecasting by comparing its different incorporation methods into neural networks. Also, we emphasize the crucial importance of selecting appropriate domain knowledge to simulate potential epidemic transmission within actual circumstances.
(4) We conduct extensive experiments to validate the performance of MPSTAN on two datasets with different epidemiological evolutionary trends. The results show that MPSTAN has accurate short- and long-term forecasting and has the generalization ability for different epidemic evolutions.

The remainder of this paper is structured as follows: In Section 2, we introduce the related work. Section 3 describes the detailed design of our proposed model. Section 4 demonstrates the experimental results and provides an analysis of the findings. Finally, a summary of the entire work is presented in Section 5.

## 2. Related Work

Many methods have been proposed for epidemic forecasting and are divided into four types of methods: traditional mathematical models, time-series models, traditional spatio–temporal models, and domain-knowledge-based spatio–temporal models.

Traditional mathematical models: Early researchers used epidemic transmission models or traditional time-series models to predict future epidemic trends. Ref. [33] uses a SIR model to predict epidemics and points out that a simple SIR model is not consistent with epidemic characteristics. Refs. [8,34] propose a series of variant models based on the SIR model to better adapt to complex and variable epidemic transmission. In addition, traditional time-series models can be used directly for epidemic forecasting due to the time-series nature of the data. Ref. [35] predicts the prevalence and incidence of epidemics by ARIMA. Ref. [10] utilizes SVR to fit the epidemiological data, but the presence of numerous spikes in daily data resulted in poor fitting. The advantages of these methods lie in their simple structure and low computational cost, but this also means that it is difficult to effectively extract potentially complex nonlinear dynamics.

Time-series models: Deep learning is widely used in time-series forecasting due to its powerful nonlinear mapping capability, where RNN and its variants LSTM and GRU are frequently applied to capture temporal dependence. Refs. [36,37] consider epidemic forecasting as a time-series forecasting problem and mainly use LSTM and its variants for epidemic forecasting, while [38] proposes a two-branch LSTM to aggregate different levels of epidemiological information. An attention mechanism is also commonly used for time-series forecasting, such as [39], which proposes a transformer-based model to predict the change in influenza cases and designs a new loss function to avoid the performance degradation of the target value. In addition, ref. [40] combines a transformer with LSTM for effective short- and long-term epidemic forecasting. Time-series forecasting models typically take into account only time dependence without considering spatial dependence. However, in the case of epidemic transmission, such models ignore the effect of inter-patch interactions on epidemic evolution. Thus, relying on temporal dependence alone can lead to inaccurate epidemic forecasting.

Traditional spatio–temporal models: Numerous studies have indicated that graph convolutional networks (GCNs) show superior results for processing data with spatial structures [41,42], and epidemic transmission can automatically be translated into a graph structure due to its spatial nature [43,44]. Ref. [18] uses time-series data as input to a GCN for epidemic forecasting. Ref. [19] proposes a dynamic location-aware attention mechanism to capture the spatial relationships between patches. Furthermore, ref. [20] fuses multimodal information in a spatio–temporal model to explore regional correlations in the epidemic transmission process. Due to the inherent nature of spatio–temporal features, models from other domains can also be applied to epidemic forecasting, such as [23], which proposes adaptive adjacency matrices to learn the relationships between nodes in a graph; ref. [45] chooses to model the temporal and spatial dimensions in parallel since the complex mapping of serial neural network structures may cause the original spatio–temporal relationships to change; [46] combines neural ODE with GCN and proposes a tensor-based model that models the spatio–temporal dependencies simultaneously to avoid limiting the model representation capability. Nevertheless, traditional spatio–temporal models lacking physical information have difficultly fitting the potentially complex dynamics [47].

Domain-knowledge-based spatio–temporal models: Several works have incorporated domain knowledge from epidemiology into neural networks. Ref. [29] utilizes a spatio–temporal model to predict the infection rates and combines it with a SIR model to predict infected cases. Ref. [30] constructs a physically guided dynamic constraint model that uses the SIR model to constrain the propagation dynamics in neural network forecasting. This dynamic constraint is based on the infection and recovery rates as well as the previous moment data to recursively derive the predicted values. Moreover, ref. [31] proposes a causal encoder–decoder structure based on the SIRD model that applies not only to the loss function but also iteratively for model construction. However, this domain knowledge (SIRD model) neglects the interactions between patches. Additionally, ref. [32] combines population mobility data to construct a metapopulation epidemic transmission model and incorporates the domain model into a neural network to help learn potential epidemic transmission dynamics. However, it is worth noting that the accuracy and completeness of mobility data can significantly affect its performance.

## 3. Methodology

In this section, we first give the problem description for epidemic forecasting. Then, we present an overview of the proposed model and details of the modules.

### 3.1. Problem Description

We use the graph G(V,E) to represent a spatial network, where V denotes the set of *N* patches, and E denotes the set of edges between patches. The adjacency matrix $A\in R^{N\times N}$ represents the connections between patches. In particular, we construct the adjacency matrix by using the gravity model [48]. The edge weight $w_{ij}$ between patches *i* and *j* is defined as:

(1) $$\begin{array}{c} w_{ij}=p_{i}^{\alpha_{1}}p_{j}^{\alpha_{2}}e^{-\frac{d_{ij}}{r}}, \end{array}$$

where $p_{i}$($p_{j}$) denotes the population size of patches *i*(*j*), $d_{ij}$ denotes the distance between patches *i* and *j*, and $\alpha_{1}$, $\alpha_{2}$, *r* are the hyperparameters. The equation indicates that if there is a high population size and close distance between a pair of patches, there is a stronger correlation of epidemic propagation between the patches. We further select the maximum *E* edge weights for all patches to make the adjacency matrix sparse and thus reduce the computational complexity. If $w_{ij}$ belongs to the set of maximum *E* edge weights of patch *i*, $A_{ij}=1$; otherwise, $A_{ij}=0$.

We use $X=\left[X_{1},X_{2},...,X_{T}\right]\in R^{N\times T\times C}$ to denote the spatio–temporal graph feature matrix, where $X_{t}$, $t\in \left[1,T\right]$ is the graph feature matrix at time step *t*, and *C* is the number of node features. Here, node features include the number of daily active cases, daily recovered cases, and daily susceptible cases. For epidemic forecasting, our goal is to learn a function f(·) that uses the adjacency matrix *A* and the node feature matrix $X_{t-T:t}$ of historical *T* time steps as inputs to predict the number of daily active cases $Y_{t+1:t+T^{\prime }}$ of future $T^{\prime }$ time steps. The problem can be formulated as follows:

(2) $$\begin{array}{c} \left[X_{t-T+1},X_{t-T+2},...,X_{t};A\right]\overset{f(\cdot )}{\to }\left[Y_{t+1},Y_{t+2}...,Y_{t+T^{\prime }}\right]. \end{array}$$

### 3.2. Model Overview

**Figure 2:**
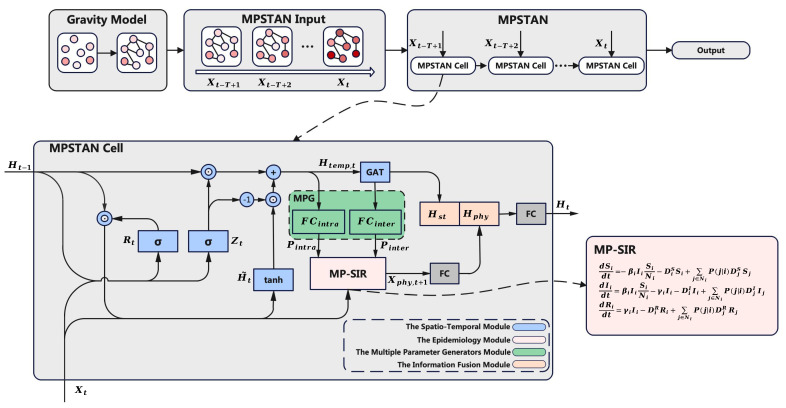
The framework of the MPSTAN model.

The overall framework of the MPSTAN model is shown in Figure 2. The model consists of a recurrent architecture, and each model cell contains four modules: namely, the spatio–temporal module, the epidemiology module, the multiple parameter generator module, and the information fusion module. At first, we use the spatio–temporal module to learn the spatio–temporal information from the input data. The learned spatio–temporal information is then passed into the parameter generation module to learn the epidemiological parameters for the epidemiological model. Further, the input and the learned parameters are passed into the epidemiological module to achieve epidemic forecasting. Finally, the learned spatio–temporal information is fused with the physical forecasting information in the information fusion module, and the output containing the fused information is passed to the MPSTAN cell at the next time step.

### 3.3. The Spatio–Temporal Module

The spatio–temporal module uses the spatio–temporal feature matrix $X\in R^{N\times T\times C}$ and the adjacency matrix $A\in R^{N\times N}$ to learn the spatio–temporal information of the epidemic data. This module embeds a graph attention network (GAT) into a gated recurrent unit (GRU), which learn the spatial dependence and the temporal dependence, respectively.

#### 3.3.1. Temporal Embedding

Initially, the GRU was widely used for time-series forecasting due to its ability to efficiently model time series; thus, we use the GRU to learn the temporal embedding of each patch. In the GRU, $Z_{t}$, $R_{t}$ denote the update gate and reset gate, respectively, at time step *t*, ${\tilde{H}}_{t}$ denotes the hidden embedding at time step *t*, $H_{t-1}$ denotes the output of the MPSTAN cell at time step t−1, and $H_{temp,t}$ denotes the output containing the temporal dependence at time step *t*:

(3) $$\begin{array}{cc} \; & Z_{t}=\sigma \left(W_{z}X_{t}+U_{z}H_{t-1}+b_{z}\right), \end{array}$$

(4) $$\begin{array}{cc} \; & R_{t}=\sigma \left(W_{r}X_{t}+U_{r}H_{t-1}+b_{r}\right), \end{array}$$

(5) $$\begin{array}{cc} \; & {\tilde{H}}_{t}=\tanh\left(W_{h}X_{t}+U_{h}\left(R_{t}⊙H_{t-1}\right)+b_{h}\right), \end{array}$$

(6) $$\begin{array}{cc} \; & H_{temp,t}=Z_{t}⊙H_{t-1}+\left(1-Z_{t}\right)⊙\tilde{H_{t}}, \end{array}$$

where ⊙ denotes element-wise multiplication, and $W_{z}$, $W_{r}$, $W_{h}$, $U_{z}$, $U_{r}$, $U_{h}$, $b_{z}$, $b_{r}$, $b_{h}$ denote the learnable parameters.

#### 3.3.2. Spatial Embedding

The epidemic evolution of each patch is not independent but is influenced by other patches at the spatial level. This is similar to GAT, which combines an attention mechanism to aggregate information from neighbor patches and update the embedding for each patch. Therefore, we use a two-layer multi-head GAT to capture the spatial dependence of epidemic evolution among patches. Firstly, we take the embedding of each patch as input and use the multi-head mechanism to compute *K* independent attention weights. The attention weight $e_{ij}^{k}$ between patch *i* and patch *j* at the *k*-th head is given by

(7) $$\begin{array}{c} e_{ij}^{k}=\sigma \left(W_{att}^{k}\left(\left(W_{temp}^{k}H_{temp,t}^{i}\right)\|\left(W_{temp}^{k}H_{temp,t}^{j}\right)\right)\right), \end{array}$$

where $W_{att}^{k}$, $W_{temp}^{k}$ denote the learnable parameters of the *k*-th head, (·‖·) denotes the vector concatenation, σ denotes the nonlinear activation function, and $e_{ij}^{k}$ omits the subscript *t*.

Then, we use the softmax function to calculate the attention scores of all the edges. The attention score between patch *i* and patch *j* at the *k*-th head as $a_{ij}^{k}$ is expressed as:

(8) $$\begin{array}{c} a_{ij}^{k}=Softmax\left(e_{ij}^{k}\right). \end{array}$$

Finally, the attention scores are used to aggregate the information from neighboring patches and update the patch embeddings $H_{st}\in R^{N\times D_{st}}$, where $D_{st}$ denotes the embedding dimension of each patch. The embedding of patch *i* as $H_{st}^{i}$ is calculated as:

(9) $$\begin{array}{c} H_{st}^{i}=\frac{1}{K}\sum_{k=1}^{K}\sum_{j\in N_{i}}a_{ij}^{k}W_{temp}^{k}H_{temp,t}^{j}, \end{array}$$

where $N_{i}$ denotes the set of neighbors of patch *i*. If $A_{ij}=1$, it indicates that patch *j* belongs to the set of neighbors of patch *i*.

### 3.4. The Epidemiology Module

We observe that the results of epidemic forecasting using only spatio–temporal models are not accurate and stable, and it is also very challenging to predict for datasets with different epidemiological evolution trends (e.g., outbreak and outbreak under control) [26]. Therefore, some works choose to use epidemiological domain knowledge to help model training, such as [30,31]. These works mainly use compartmental models as domain knowledge, such as the SIR model. The SIR model is the most typical model in epidemic transmission, where S denotes the susceptible individuals, I denotes the infected individuals, and R denotes the recovered individuals. The model uses three differential equations to represent the changes to the three state populations in patch *i*:

(10) $$\begin{array}{cc} \; & \frac{dS_{i}}{dt}=-\beta_{i}I_{i}\frac{S_{i}}{N_{i}}, \end{array}$$

(11) $$\begin{array}{cc} \; & \frac{dI_{i}}{dt}=\beta_{i}I_{i}\frac{S_{i}}{N_{i}}-\gamma_{i}I_{i}, \end{array}$$

(12) $$\begin{array}{cc} \; & \frac{dR_{i}}{dt}=\gamma_{i}I_{i}, \end{array}$$

where $\beta_{i}$ and $\gamma_{i}$ denote the infection and recovery rates, respectively, of epidemic transmission in patch $i\in \left[1,\cdots ,N\right]$. However, the SIR model is limited to simulate epidemic transmission within a single patch and neglects the inter-patch interactions. Therefore, ref. [32] uses population mobility data to construct a metapopulation epidemic model and iteratively calculates the daily confirmed cases using neural networks. In addition, other mobility change data (e.g., GPS trajectory data) can also be used to construct a metapopulation epidemic model. However, accurate collection of mobility data is challenging, and other data may not fully reflect actual population mobility patterns.

To overcome the limitation of data availability, we develop an adaptive approach to define inter-patch interactions and construct a metapopulation epidemic model named the metapopulation-based SIR (MP-SIR) model that does not rely on mobility data. The MP-SIR model is based on the original SIR model with inter-patch mobility parameters to represent the mobility of populations at each state between patches:

(13) $$\begin{array}{cc} \; & \frac{dS_{i}}{dt}=-\beta_{i}I_{i}\frac{S_{i}}{N_{i}}-D_{i}^{S}S_{i}+\sum_{j\in N_{i}}P\left(j|i\right)D_{j}^{S}S_{j}, \end{array}$$

(14) $$\begin{array}{cc} \; & \frac{dI_{i}}{dt}=\beta_{i}I_{i}\frac{S_{i}}{N_{i}}-\gamma_{i}I_{i}-D_{i}^{I}I_{i}+\sum_{j\in N_{i}}P\left(j|i\right)D_{j}^{I}I_{j}, \end{array}$$

(15) $$\begin{array}{cc} \; & \frac{dR_{i}}{dt}=\gamma_{i}I_{i}-D_{i}^{R}R_{i}+\sum_{j\in N_{i}}P\left(j|i\right)D_{j}^{R}R_{j}, \end{array}$$

where P(j∣i) denotes the mobility probability of patch *j* to patch *i*, and $D_{i}^{S}$, $D_{i}^{I}$, $D_{i}^{R}$ denote the mobility rates of susceptible, infected, and recovered individuals, respectively, in patch *i*.

Taking Equation (14) as an example, the change in the number of infected individuals within patch *i* is affected by four possible events: (i) susceptible individuals $S_{i}$ become infected with probability $\beta_{i}$ after contact with infected individuals $I_{i}$; (ii) infected individuals $I_{i}$ recover with probability $\gamma_{i}$; (iii) infected individuals $I_{i}$ within patch *i* move to other patches with the mobility rate $D_{i}^{I}$; (iv) infected individuals $I_{j}$ from patch *j* move toward patch *i* with the mobility rate $D_{j}^{I}$. We simply assume that the probability of a patch migrating to other neighboring patches is equal. Formally, the mobility probability of patch *j* to patch *i* P(j∣i) is computed as follows:

(16) $$\begin{array}{c} P\left(j|i\right)=\frac{1}{\left|N_{j}\right|}. \end{array}$$

Equation (16) is a simplification in the absence of mobility data. If such data are available, they can be utilized to more accurately estimate the migration probability between patches, as in [15] and as shown in Equation (17):

(17) $$\begin{array}{c} P\left(j|i\right)=\frac{T_{ji}}{\sum_{l\in N_{j}}T_{jl}}, \end{array}$$

where $T_{ji}$ denotes the population mobility data of patch *j* moving to patch *i*, and *l* denotes the neighboring patches of patch *j*.

We use neural networks to generate intra- and inter-patch MP-SIR model parameters $P_{intra}=\left[\beta ,\gamma \right]\in R^{N\times 2}$, $P_{inter}=\left[D^{S},D^{I},D^{R}\right]\in R^{N\times 3}$, and we describe them in detail in Section 3.5. Finally, the epidemic data and the generated MP-SIR model parameters are used as inputs to the MP-SIR model for domain-knowledge-based epidemic forecasting:

(18) $$\begin{array}{cc} \; & \Delta X_{phy,t}=MP-SIR\left(X_{t},P_{intra},P_{inter}\right), \end{array}$$

(19) $$\begin{array}{cc} \; & X_{phy,t+1}=X_{t}+\Delta X_{phy,t}, \end{array}$$

where $\Delta X_{phy,t}\in R^{N\times 3}$ denotes the change in the number of individuals in each state at time step *t*, and $X_{phy,t+1}=\left[X_{phy,t+1}^{S},X_{phy,t+1}^{I},X_{phy,t+1}^{R}\right]\in R^{N\times 3}$ denotes epidemic forecasting at time step t+1.

This model applies to various diseases that propagate within spatio–temporal ranges, such as influenza, COVID-19, and others. However, the model has certain data requirements, which involve gathering information on the cases of infected, susceptible, and recovered individuals across various regions impacted by the epidemic. These data serve as the foundation for constructing a metapopulation-based epidemic transmission model (MP-SIR) used for spatio–temporal epidemic forecasting.

### 3.5. The Multiple Parameter Generator Module

We use embeddings containing different information to learn intra- and inter-patch physical model parameters $P_{intra}\in R^{N\times 2}$, $P_{inter}\in R^{N\times 3}$ separately instead of directly by using embeddings containing spatio–temporal information. The intra-patch physical model parameters β, γ indicate the epidemic evolution within a single patch and are mainly affected by the temporal dependence, while the inter-patch physical model parameters $D^{S}$, $D^{I}$, $D^{R}$ indicate the inter-patch population mobility and are mainly affected by the spatio–temporal dependence. Therefore, we generate these two types of physical model parameters by passing embeddings containing only the temporal dependence and the spatio–temporal dependence to the two fully connected layers, respectively:

(20) $$\begin{array}{cc} P_{intra} & =FC_{intra}\left(H_{temp,t}\right), \end{array}$$

(21) $$\begin{array}{cc} P_{inter} & =FC_{inter}\left(H_{st}\right). \end{array}$$

### 3.6. The Information Fusion Module

In this module, the information between neural network forecasting $H_{st}\in R^{N\times D_{st}}$ and physical model forecasting $X_{phy,t+1}\in R^{N\times 3}$ is fused. First, we map $X_{phy,t+1}$ to $H_{phy}\in R^{N\times D_{st}}$ using a fully connected layer that aims to keep the physical forecasting with the same dimensions as the neural network forecasting:

(22) $$\begin{array}{c} H_{phy}=FC\left(X_{phy,t+1}\right). \end{array}$$

Next, the neural network forecasting is concatenated with the physical forecasting. Finally, a fully connected layer is used to generate the final output $H_{t}\in R^{N\times D_{gru}}$ of the MPSTAN cell at time step *t*, where $D_{gru}$ denotes the dimensions of the GRU:

(23) $$\begin{array}{c} H_{t}=FC\left(H_{st}\;\|\;H_{phy}\right). \end{array}$$

### 3.7. Output Layer

The output of the MPSTAN model is divided into two parts: neural network forecasting and physical model forecasting.

Neural network forecasting:

We use the final output $H_{T}\in R^{N\times D_{gru}}$ of MPSTAN as the input of a fully connected layer to predict the number of infected individuals $Y^{st}\in R^{N\times T^{\prime }}$ in all patches for the next $T^{\prime }$ time steps:

(24) $$\begin{array}{c} Y^{st}=FC_{pred}\left(H_{T}\right). \end{array}$$

Physical model forecasting:

The input data from the last day and the final trained model parameters are used as inputs for the MP-SIR model to recursively predict the number of infected individuals $Y^{phy}\in R^{N\times T^{\prime }}$ in all patches for the next $T^{\prime }$ time steps:

(25) $$\begin{array}{cc} \; & \Delta X_{phy,T}=MP-SIR\left(X_{T},P_{intra,T},P_{inter,T}\right), \end{array}$$

(26) $$\begin{array}{cc} \; & X_{phy,T+1}=X_{T}+\Delta X_{phy,T},\\ \; & \cdots \end{array}$$

(27) $$\begin{array}{cc} \; & Y^{phy}=\left[X_{phy,T+1}^{I},X_{phy,T+2}^{I},\cdots ,X_{phy,T+T^{\prime }}^{I}\right]. \end{array}$$

### 3.8. Optimization

We utilize epidemiological domain knowledge for model construction and loss functions to more effectively help MPSTAN models learn the epidemiological evolution trends. We compare the predicted values $Y^{st}$, $Y^{phy}$ of neural networks and physical models with the ground truth $\hat{Y}$ and then optimize MAE loss via gradient descent:

(28) $$\begin{array}{c} L\left(\Theta \right)=\frac{1}{N\times T^{\prime }}\sum_{i=1}^{N}\sum_{\tau =1}^{T^{\prime }}\left(\left|Y_{i,\tau }^{st}-{\hat{Y}}_{i,\tau }\right|+\left|Y_{i,\tau }^{phy}-{\hat{Y}}_{i,\tau }\right|\right). \end{array}$$

The design of this loss function is inspired by physics-informed neural networks (PINNs) [49], which emphasize the introduction of physical information constraints during training, enabling the model to learn with fewer data samples and to better conform to specific physical rules within a given domain.

## 4. Experiments

### 4.1. Datasets

**Figure 3:**
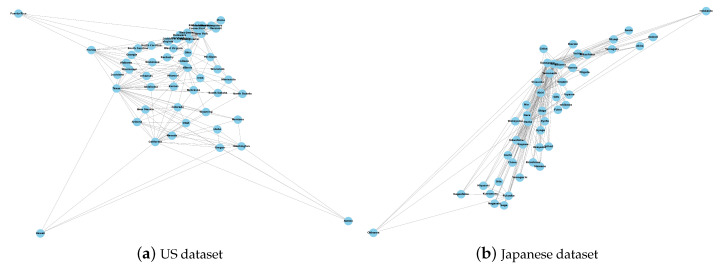
The interaction graphs between patches from two datasets.

Our experiment mainly involves two types of data. The first type is the real COVID-19 datasets from the United States and Japan. The second type is the information data of patches, such as population and distance, which will be used in the gravity model to generate the graph. As shown in Table 1, the US dataset is state-level data collected from the Johns Hopkins University Coronavirus Resource Center [24] and provides the number of daily active cases, daily recovered cases, daily susceptible cases and total population for 52 states from 1 May 2020 to 31 December 2020 (245 days). The Japanese dataset is prefecture-level data collected from the Japan LIVE Dashboard [25], which provides the number of daily active cases, daily recovered cases, daily susceptible cases, and total population for 47 prefectures from 15 January 2022 to 14 June 2022 (151 days). In temporal order, we divide each patch of these two datasets into training, validation, and test sets at ratios of 60%, 20%, and 20%, respectively, and normalize all data to the range (0, 1). As pointed out in references [50,51], epidemic forecasting often overlooks undocumented cases, and the quality of estimated data impacts subsequent forecasting. This study primarily focuses on analyzing model forecasting accuracy assuming that these data are ideal. Based on the gravity model described in Equation (1), we use the population and distance data of patches to generate adjacency matrices for each COVID-19 dataset. A simple visualization of the interaction graphs is shown in Figure 3.

### 4.2. Experimental Details

#### 4.2.1. Baselines

We compare our model with the following five kinds of baselines: (i) traditional mathematical models: SIR and ARIMA; (ii) time-series models based on recurrent structure: GRU; (iii) traditional spatio–temporal models: GraphWaveNet, STGODE, CovidGNN, and ColaGNN; (iv) domain-knowledge-based spatio–temporal model: STAN; (v) two time-series models based on transformers: PatchTST and Crossformer. All baseline models use daily active cases, daily susceptible cases, and daily recovered cases as inputs to predict future daily active cases.

(1) **SIR** [5]: The SIR model uses three differential equations to calculate the change in the number of susceptible, infected, and recovered cases in a single patch.
(2) **ARIMA** [35]: The auto-regressive integrated moving average model is widely used for time-series forecasting. We use ARIMA to predict daily active cases for each patch.
(3) **GRU** [12]: The gated recurrent unit is a variant of RNN that uses fewer parameters to implement the gating mechanism compared to LSTM. We use a GRU for each patch separately to predict daily active cases.
(4) **GraphWaveNet** [23]: GraphWaveNet combines an adaptive adjacency matrix, diffusion convolution, and gated TCN to capture spatio–temporal dependencies.
(5) **STGODE** [46]: STGODE proposes a spatio–temporal tensor model by combining neural ODE with GCN to achieve unified modeling of spatio–temporal dependencies.
(6) **CovidGNN** [18]: CovidGNN uses the time-series of each patch as node features and predicts epidemics using GCN with skip connections.
(7) **ColaGNN** [19]: ColaGNN designs a dynamic adjacency matrix using an attention mechanism and adopts a multi-scale dilated convolutional layer for long- and short-term epidemic forecasting.
(8) **STAN** [30]: STAN utilizes the gravity model to construct networks and applies epidemiological domain knowledge to the loss function, which specifically constructs a dynamics constraint loss by combining with the SIR model.
(9) **PatchTST** [52]: Based on the transformer, PatchTST considers the channel independence of input features and uses a patching mechanism to extract local semantic information from time-series data.
(10) **Crossformer** [53]: Based on the transformer, Crossformer introduces a two-stage attention mechanism to effectively capture dependencies across both the time and feature dimensions.

#### 4.2.2. Settings

To verify the effectiveness of the model for short- and long-term forecasting, we set the input time length as 5, the forecasting time length as 5 and 10 for short-term forecasting, and the forecasting time length as 15 and 20 for long-term forecasting. For various forecasting tasks on different datasets, we conduct multiple independent experiments and average the results to reduce randomness. In the model, the dimensions of GRU and GAT are set to 64 and 32, respectively. Further, the number of heads K in GAT is set to 2. The settings of the hyperparameters for the gravity model are based on the settings in [30], where $\alpha_{1}$ = 0.1, $\alpha_{2}$ = 0.1, and *r* = 1 × 10^4^. We set the number of epochs to 50 and use the Adam optimizer with a learning rate of 1e-3.

#### 4.2.3. Evaluation Metrics

In this study, we choose the mean absolute error (MAE), root mean squared error (RMSE), mean absolute percentage error (MAPE), Pearson’s correlation coefficient (PCC), and concordance correlation coefficient (CCC) to evaluate the performance of each model, where lower MAE, RMSE, and MAPE vales and higher PCC and CCC values indicate better forecasting performance. The above evaluation metrics are expressed as follows:

(29) $$\begin{array}{cc} \; & MAE=\frac{1}{N\times T^{\prime }}\sum_{i=1}^{N}\sum_{\tau =1}^{T^{\prime }}\left(\right|Y_{i,\tau }^{st}-{\hat{Y}}_{i,\tau }\left|\right), \end{array}$$

(30) $$\begin{array}{cc} \; & RMSE=\sqrt{\frac{1}{N\times T^{\prime }}\sum_{i=1}^{N}\sum_{\tau =1}^{T^{\prime }}\left(\right|Y_{i,\tau }^{st}-{\hat{Y}}_{i,\tau }{\left|\right)}^{2}}, \end{array}$$

(31) $$\begin{array}{cc} \; & MAPE=\frac{100\%}{N\times T^{\prime }}\sum_{i=1}^{N}\sum_{\tau =1}^{T^{\prime }}\left|\frac{Y_{i,\tau }^{st}-{\hat{Y}}_{i,\tau }}{{\hat{Y}}_{i,\tau }}\right|, \end{array}$$

(32) $$\begin{array}{cc} \; & PCC=\frac{\sum \left(Y_{i,\tau }^{st}-{\bar{Y}}_{i,\tau }^{st}\right)\left({\hat{Y}}_{i,\tau }-{\bar{\hat{Y}}}_{i,\tau }\right)}{\sqrt{\sum {\left(Y_{i,\tau }^{st}-{\bar{Y}}_{i,\tau }^{st}\right)}^{2}\sum {\left({\hat{Y}}_{i,\tau }-{\bar{\hat{Y}}}_{i,\tau }\right)}^{2}}}, \end{array}$$

(33) $$\begin{array}{cc} \; & CCC=\frac{2\rho \sigma_{x}\sigma_{y}}{\sigma_{x}^{2}+\sigma_{y}^{2}+{\left(\mu_{x}-\mu_{y}\right)}^{2}}, \end{array}$$

where ρ denotes the correlation coefficient between the two variables, $\mu_{x}$ and $\mu_{y}$ denote the mean of the two variables, $\sigma_{x}^{2}$, $\sigma_{y}^{2}$ are the corresponding variances, and ∑ is an abbreviation of $\sum_{i=1}^{N}\sum_{\tau =1}^{T^{\prime }}$.

### 4.3. Forecasting Performance

As shown in Table 2 and Table 3, we evaluate the performance of our method with all the baselines on the US dataset and the Japanese dataset, respectively, for predicting daily active cases, where bold and underlined indicate optimal and suboptimal, respectively, and ’Improvement’ denotes the improved rate of MPSTAN compared to the suboptimal forecasting results. On the US dataset, our method achieves state-of-the-art (SOTA) performance for both short-term (T = 5, 10 days) and long-term (T = 15, 20 days) forecasting. In particular, our forecasting results for all the forecasting tasks show significant improvements over the suboptimal forecasting, where MAE improves at least 4.01%, RMSE improves at least 7.64%, MAPE improves at least 4.61%, PCC improves at least 0.31%, and CCC improves at least 0.11%. While our method may not fully achieve SOTA performance on the Japanese dataset, it can achieve optimal or competitive forecasting results compared to other models, demonstrating strong competitiveness, where MAE improves at least 11.43%, RMSE improves at least 21.32%, MAPE improves at least 24.81%, and CCC improves at least 3.32%. In summary, compared to all baseline models, MPSTAN can provide more accurate and stable forecasting for different real-world epidemic datasets.

Next, we discuss specifically the performance comparison between different models. Traditional mathematical models (e.g., SIR and ARIMA) often outperform neural network models in short-term forecasting, but the performance becomes worse in long-term forecasting. This may be because the predictive accuracy of traditional mathematical models is highly dependent on the time length, and long-term forecasting requires more historical data. Insufficient historical data can lead to forecasting errors, and the cumulative effect of errors increases with longer forecasting times, resulting in worse long-term forecasting results.

In addition, we observe that traffic flow models, particularly the STGODE, face challenges with providing stable and accurate forecasting for different tasks. This may be attributed to the fact that epidemic data are sparser and noisier than traffic flow data, increasing the likelihood of these models overfitting when applied to epidemic data. Through observation, it is noticed that the ColaGNN model also faces difficulties with providing accurate forecasting. The poorer performance of ColaGNN may be because the ColaGNN model does not incorporate a domain model, which leads to its poor performance when handling these datasets.

By comparing MPSTAN with domain-knowledge-based models (e.g., STAN), the results show that MPSTAN performs better than STAN, highlighting the effectiveness of this integrated neural network framework in achieving more accurate forecasting by introducing epidemiological domain knowledge. This framework involves two main aspects: integrating domain knowledge into the deep learning framework and modeling metapopulation transmission. Furthermore, in Section 4.4, we discuss the impact of these two aspects on forecasting results, including the effects of integration methods and inter-patch interactions.

Finally, we compare MPSTAN with the latest SOTA time-series models based on the transformer (e.g., PatchTST and Crossformer). It is evident that the PatchTST model performs well in epidemic forecasting, whereas the Crossformer model exhibits relatively poor performance. Overall, MPSTAN consistently provides more accurate or competitive forecasting compared to the latest transformer models.

### 4.4. Ablation Study

To explore the impact of epidemiological domain knowledge on epidemic forecasting and to verify the effectiveness of the model components, we further conduct ablation experiments on the US and Japanese datasets.

(1) **MPSTAN w/o Phy-All**: Remove epidemiological domain knowledge from both model construction and the loss function. We use only the spatio–temporal module for epidemic forecasting.
(2) **MPSTAN w/o Phy-Loss**: Remove epidemiological domain knowledge from the loss function. We only implement the knowledge in model construction.
(3) **MPSTAN w/o Phy-Model**: Remove the epidemiological domain knowledge from model construction. We predict physical model parameters in the output layer and implement the knowledge in the loss function.
(4) **MPSTAN w/o Mobility**: Combine epidemiological domain knowledge without considering population mobility into the model—mainly by using the SIR model instead of the MP-SIR model.
(5) **MPSTAN w/o MPG**: Remove multiple parameter generators (MPGs). We generate all the physical model parameters using a single parameter generator for embeddings containing spatio–temporal information.

The results of the ablation experiments are shown in Table 4 and Table 5, where bold indicates better performance for the ablation model or MPSTAN. Firstly, we analyze the effectiveness of domain knowledge in epidemic forecasting by comparing the performance of MPSTAN with MPSTAN w/o Phy-All on two datasets. The results show that the MPSTAN w/o Phy-All model, which lacks domain knowledge, performs extremely poorly in epidemic forecasting, highlighting the crucial role of epidemiological domain knowledge in epidemic forecasting.

To further investigate the impact on epidemic forecasting of different methods for integrating domain knowledge, we compare MPSTAN w/o Phy-Loss and MPSTAN w/o Phy-Model with MPSTAN. On the US dataset, MPSTAN, which applies domain knowledge to both model construction and the loss function, can more accurately predict epidemic trends, as shown in Table 4. In Table 5, for short-term forecasting on the Japanese dataset, MPSTAN performs worse than MPSTAN w/o Phy-Loss, which applies domain knowledge only to model construction, but it still provides competitive forecasting. In long-term forecasting, MPSTAN outperforms the other two models. Overall, incorporating domain knowledge into both model construction and the loss function can better help the model learn the basic dynamics of epidemic transmission and improve forecasting accuracy. By comparing MPSTAN w/o Phy-Loss and MPSTAN w/o Phy-Model using two datasets, we find that the former performs better in all forecasting tasks, indicating that applying domain knowledge to model construction is more beneficial for accurate epidemic forecasting than applying it to the loss function. In addition, by comparing MPSTAN w/o Phy-All and MPSTAN w/o Phy-Model, we find that using domain knowledge to only constrain the loss function may lead to poorer forecasting performance. Therefore, we believe that incorporating domain knowledge into model construction is essential, and simultaneously applying it to the loss function can improve the predictive accuracy of the model.

For the remaining model components, the effectiveness of the establishment of the metapopulation model and multiple parameter generators can be verified by using MPSTAN w/o Mobility and MPSTAN w/o MPG, respectively. On the US dataset, MPSTAN outperforms MPSTAN w/o Mobility for forecasting tasks with T = 5, 10, and 15 days. However, the opposite result is observed for the T = 20 task, which may be due to the fact that inter-patch physical parameters are no longer sufficient to define the population mobility when the forecasting time is longer. Overall, MP-SIR, a metapopulation epidemic model that considers population mobility, is more beneficial for model training than traditional SIR. Additionally, comparing MPSTAN with MPSTAN w/o MPG reveals that using only one parameter generator to generate all physical model parameters may lead to poorer predictive performance.

**Figure 4:**
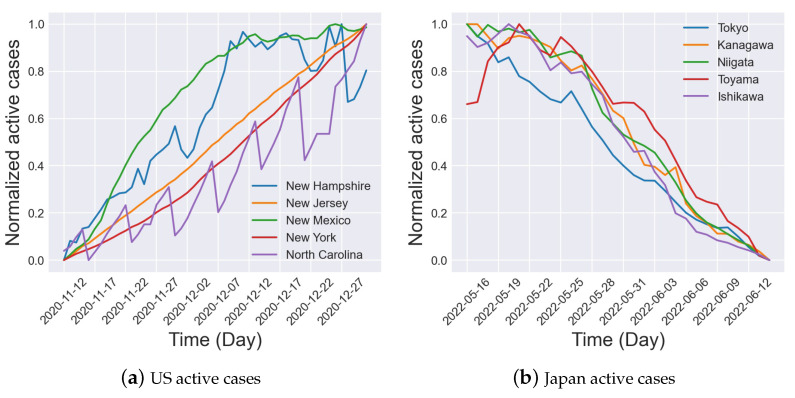
Samples of typical cities in the US and Japanese datasets.

On the Japanese dataset, we observe that the performance of MPSTAN w/o Mobility and MPSTAN w/o MPG is mostly superior to MPSTAN. We believe that this is due to the fact that these two datasets are collected at different times and locations, leading to differences in disease control measures and public awareness. To confirm this, we randomly select five cities from each dataset and display the normalized daily active cases of these cities in Figure 4. The figure clearly shows that US cities experienced a surge in active cases, while Japanese cities effectively controlled the spread of the disease, resulting in a decrease in active cases. Moreover, we investigate the COVID-19 Community Mobility Reports [54] from Google for the corresponding time periods of these two datasets. We observe that the park population movement in the US is higher than the pre-epidemic baseline, while in Japan it is lower than the baseline. Possible reasons for the above situation could be that the data collected in the United States are from an earlier period when the COVID-19 prevention and control policies were possibly more relaxed, resulting in greater population mobility. However, park movement does not necessarily reflect typical mobility patterns. Moreover, the data collected in Japan are from a later period when more comprehensive measures had been implemented, and the public may have become more aware of the importance of self-isolation, leading to lower population mobility. Therefore, MPSTAN may not be fully applicable to the Japanese dataset, possibly due to different restrictions at this stage leading to a decrease in population mobility. Consequently, the traditional SIR model is more suitable than the MP-SIR model. The multiple parameter generators (MPGs) are essentially based on the metapopulation epidemic model, and thus, the forecasting accuracy of MPSTAN w/o MPG is higher.

Furthermore, we recognize that no single source of domain knowledge can be universally applied to all complex epidemic data. Thus, when selecting domain knowledge to integrate into neural networks, it is necessary to consider the actual circumstances and choose more representative knowledge to achieve more accurate forecasting.

### 4.5. Effect of Hyperparameters

In this section, we study the effect of hyperparameters on performance, focusing on the dimensions of the GRU and GAT. We vary one parameter at a time while keeping the other parameters constant. In addition, the dimension range is set to [8, 16, 32, 64, 128], T = 5 days is selected as the task on the US dataset, while MAE, RMSE, and MAPE are chosen as the evaluation metrics.

**Figure 5:**
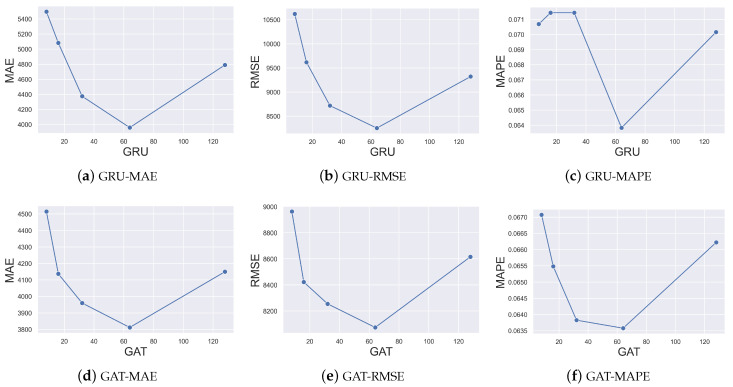
Effect of hyperparameters on performance.

Figure 5 shows the effects of different dimensions of the GRU and GAT on the performance. It can be seen that the forecasting performance is poor when the number of dimensions is small, and it gradually becomes better when the number of dimensions increases, which is because more parameters are involved in fitting the potential dynamics of the epidemic. When the number of dimensions continues to increase, the forecasting performance also becomes worse. The possible reason of this issue may be that the epidemic data are sparse, and an excessive number of parameters leads to overfitting.

### 4.6. Model Complexity

We analyze the model complexity by comparing the parameter number in the neural network and the training time consumed for all models at T = 5 days on the US dataset. As shown in Table 6, the number of neural network parameters in MPSTAN is significantly less than in other models. This is because MPSTAN makes extensive use of epidemiological domain knowledge (e.g., model construction and loss functions), thus reducing the reliance on neural networks and lowering the number of parameters. By comparing GRU and MSPTAN, we find that the number of parameters is similar, but the former ignores the spatial dependence and the intrinsic propagation mechanism of the epidemic, which can only be used for temporal forecasting of a single patch, while the latter perfectly solves the above problems and provides stable and accurate forecasting for different trends. Although the recurrent structure of MPSTAN involves high time costs in the iterative computation of domain models, the overall training time remains acceptable for epidemic forecasting.

## 5. Conclusions

Current spatio–temporal attention network models are primarily designed for traffic flow forecasting, with relatively fewer efforts being made in epidemic forecasting. Existing epidemic forecasting models have limitations in integrating domain knowledge, such as neglecting the mobility of populations between patches or relying on population mobility data. To overcome these limitations, this paper introduces a metapopulation-based spatio–temporal attention network (MPSTAN) for epidemic forecasting. The model uses an adaptive approach to define interactions between patches and applies the constructed domain model to model construction and the loss function of MPSTAN to better learn the underlying dynamics of epidemic propagation. Experiments show that MPSTAN outperforms other baselines and is more stable on two real datasets with different epidemiological evolution trends. Additionally, we further analyze the effectiveness of incorporating domain knowledge and find that it improves the accuracy of forecasting in the learning model. Specifically, domain knowledge plays a more critical role in model construction than loss functions, and applying it to both aspects can provide a better fit to potential epidemiological dynamics. We also recognize that no single domain knowledge source can perfectly fit epidemic forecasting in different real-world situations. Instead, we should select domain knowledge that is more representative based on the actual circumstances to achieve more accurate forecasting. We also discuss the impact of hyperparameters on the model, as excessively small or large hyperparameters can lead to underfitting or overfitting, respectively, so appropriate hyperparameters must be chosen. Finally, we analyze model complexity and find that compared to all baselines, MPSTAN requires fewer neural network parameters due to its greater integration of domain knowledge. Although this leads to high time costs, the overall training time remains acceptable for epidemic forecasting.

Our model achieves state-of-the-art or competitive results in epidemic forecasting for different epidemic trends, but there are still several aspects where performance can be improved. Firstly, graph construction has a significant impact on the entire learning model, as it affects the propagation of spatial information and the inter-patch interactions of the physical model. Therefore, a reasonable graph structure is crucial. Currently, we use the gravity model to construct the graph structure, which relies on prior knowledge, but it may overlook some potential information, resulting in an incomplete capture of the correct graph information between patches. In addition, the graph information between patches changes over time rather than being fixed. Hence, in the future, we will combine potential graph information to construct a dynamic graph structure to better describe interactive graphs of epidemics. Meanwhile, this model uses data from infected, recovered, and susceptible individuals for time-series forecasting, which is often available for epidemic forecasting. However, the size of undocumented cases is also crucial for prediction. Although these cases are not recorded by the health department, they may significantly impact the actual transmission rate of the disease. Therefore, incorporating these undocumented cases into forecasting could help provide understanding of the complex effects of epidemic spread. In addition, while our model provides a valuable foundational model, it lacks consideration of the impacts of non-pharmaceutical interventions and vaccination, which is a limitation of the model. It is possible to introduce the effects of vaccination, non-pharmaceutical interventions, and other measures into our model to better capture the potential dynamics of epidemic spread. Furthermore, in model construction, we currently simply connect the neural network results with domain knowledge from the physical model without considering their respective roles or weights, which may also lead to a decrease in accuracy. Therefore, we will carefully analyze the roles of the neural network and domain knowledge in epidemic forecasting and explore more effective methods to fuse the information of the two, such as introducing gating mechanisms to achieve more accurate forecasting.

## Figures and Tables

**Table 1:** Statistical information of the datasets.

Dataset	Data Level	Data Size	Time Range	Min	Max	Mean	Std
US	State-level	52×245	2020.5.1 –2020.12.31	0	838,855	40,438	75,691
Japan	Prefecture-level	47×151	2022.1.15–2022.6.14	104	198,011	11,458	21,188

**Table 2:** Performance comparison with baseline on the US dataset.

	The US dataset									
	T = 5 days					T = 10 days				
Model	MAE	RMSE	MAPE	PCC	CCC	MAE	RMSE	MAPE	PCC	CCC
SIR	5660	15,656	25.81%	99.16%	99.14%	10,608	33,766	41.94%	96.23%	96.09%
ARIMA	6475	22,095	14.01%	98.33%	98.31%	11,489	44,779	26.36%	93.66%	93.39%
GRU	18,348	32,950	21.88%	97.88%	95.63%	26,749	47,328	32.52%	95.66%	90.39%
GraphWaveNet	13,875	22,559	17.85%	99.46%	97.82%	9526	15,673	16.64%	99.21%	99.09%
STGODE	70,454	116,865	83.21%	91.95%	64.48%	53,693	83,823	63.51%	87.89%	62.19%
CovidGNN	9453	21,612	9.91%	99.07%	98.17%	16,052	37,586	15.03%	96.87%	94.00%
ColaGNN	66,005	111,622	77.57%	53.54%	41.79%	51,822	91,680	57.61%	80.18%	62.46%
STAN	10,024	19,214	17.98%	98.70%	98.65%	13,993	25,963	19.38%	97.80%	97.49%
PatchTST	5086	12,119	7.62%	99.49%	99.46%	8033	17,283	11.70%	99.02%	98.89%
Crossformer	20,469	41,348	21.99%	95.88%	93.09%	24,428	47,851	26.06%	94.64%	90.32%
MPSTAN	**3960**	**8255**	**6.38%**	**99.80%**	**99.75%**	**7711**	**14,463**	**10.73%**	**99.55%**	**99.20%**
Improvement	22.14%	31.88%	16.27%	0.31%	0.29%	4.01%	7.72%	8.29%	0.34%	0.11%
	T = 15 days					T = 20 days				
Model	MAE	RMSE	MAPE	PCC	CCC	MAE	RMSE	MAPE	PCC	CCC
SIR	16,573	60,984	57.38%	89.04%	88.26%	23,963	101,612	76.12%	76.44%	73.21%
ARIMA	17,151	74,295	43.01%	84.86%	83.59%	24,849	121,875	65.20%	69.78%	65.31%
GRU	33,968	59,804	41.21%	92.67%	83.94%	38,202	65,762	45.54%	90.44%	80.61%
GraphWaveNet	47,020	76,735	51.64%	90.76%	72.64%	48,154	82,098	51.19%	84.43%	68.47%
STGODE	72,622	117,611	107.65%	82.26%	50.75%	72,132	109,536	84.84%	85.16%	42.81%
CovidGNN	21,660	48,169	19.85%	94.68%	89.71%	26,985	57,085	24.57%	92.64%	84.95%
ColaGNN	33,419	55,424	41.36%	92.43%	79.63%	47,837	77,656	52.48%	92.49%	70.90%
STAN	16,784	33,383	20.78%	96.43%	95.49%	18,679	36,180	26.81%	96.09%	94.52%
PatchTST	**10,120**	19,986	15.39%	98.79%	98.48%	14,178	28,244	20.10%	97.67%	96.84%
Crossformer	27,084	50,297	30.01%	94.69%	88.96%	28,668	51,294	32.64%	95.10%	88.37%
MPSTAN	10,148	**18,460**	**14.68%**	**99.25%**	**98.68%**	**12,728**	**22,923**	**18.68%**	**98.81%**	**97.91%**
Improvement	-	7.64%	4.61%	0.47%	0.20%	10.23%	18.84%	7.06%	1.17%	1.10%

**Table 3:** Performance comparison with baseline on the Japanese dataset.

	The Japanese dataset									
	T = 5 days					T = 10 days				
Model	MAE	RMSE	MAPE	PCC	CCC	MAE	RMSE	MAPE	PCC	CCC
SIR	896	**1572**	18.89%	**99.11%**	**97.91%**	1703	2874	39.38%	**97.73%**	93.67%
ARIMA	1113	3137	24.33%	91.74%	91.37%	2433	8719	59.59%	63.42%	57.19%
GRU	2156	3955	58.91%	94.06%	89.02%	2702	5130	69.49%	92.33%	83.80%
GraphWaveNet	2048	4490	39.06%	94.93%	87.35%	2744	6447	48.88%	92.64%	79.24%
STGODE	5420	13057	103.14%	83.94%	57.16%	8208	18396	158.08%	85.00%	50.91%
CovidGNN	1042	2305	18.06%	97.27%	95.71%	1887	3942	39.40%	95.77%	89.48%
ColaGNN	2566	5746	50.29%	92.17%	82.16%	5294	10,402	101.50%	86.60%	63.78%
STAN	1070	2400	22.97%	95.87%	94.82%	1623	3165	34.38%	94.80%	91.97%
PatchTST	**828**	2987	**15.90%**	92.33%	91.56%	**1324**	**2608**	31.64%	95.09%	**94.09%**
Crossformer	1732	3826	34.91%	94.82%	89.70%	2741	6161	58.48%	88.98%	78.61%
MPSTAN	1016	2311	16.91%	96.74%	95.60%	1356	3016	**24.34%**	93.38%	92.27%
Improvement	-	-	-	-	-	-	-	-	-	-
Model	T = 15 days					T = 20 days				
	MAE	RMSE	MAPE	PCC	CCC	MAE	RMSE	MAPE	PCC	CCC
SIR	2632	4373	66.60%	95.22%	87.05%	3515	5883	92.93%	92.08%	79.20%
ARIMA	3443	7715	86.16%	65.62%	61.39%	3757	7513	130.90%	72.79%	66.56%
GRU	2124	3758	59.84%	88.58%	87.70%	2977	5343	68.13%	71.75%	70.72%
GraphWaveNet	2828	6520	49.39%	93.62%	79.34%	2773	6547	46.11%	**92.96%**	79.38%
STGODE	10,330	23,345	195.76%	82.22%	38.62%	12,156	27,407	221.51%	83.58%	33.33%
CovidGNN	2988	6515	66.73%	90.20%	77.42%	3990	8805	94.82%	84.97%	67.12%
ColaGNN	4192	8688	93.21%	84.31%	67.68%	7195	15,400	140.32%	84.30%	50.40%
STAN	2026	3887	51.03%	93.86%	88.92%	2804	5238	72.10%	90.59%	82.24%
PatchTST	1654	**2984**	40.45%	**95.61%**	**92.80%**	2321	5102	56.56%	87.05%	81.40%
Crossformer	3575	7896	84.07%	82.25%	69.05%	5237	14,458	115.39%	71.97%	47.94%
MPSTAN	**1465**	3104	**28.29%**	91.84%	91.29%	**1854**	**4014**	**34.67%**	85.78%	**84.97%**
Improvement	11.43%	-	30.06%	-	-	20.12%	21.32%	24.81%	-	3.32%

**Table 4:** Ablation study on the US dataset.

	The US dataset									
	T = 5 days					T = 10 days				
Model	MAE	RMSE	MAPE	PCC	CCC	MAE	RMSE	MAPE	PCC	CCC
MPSTAN w/o Phy-All	14,865	34,756	10.96%	96.19%	95.18%	22,911	54,185	17.38%	91.55%	86.63%
MPSTAN w/o Phy-Loss	18,908	39,201	14.62%	94.53%	92.97%	15,201	27,700	16.09%	98.53%	96.57%
MPSTAN w/o Phy-Model	19,002	45,127	13.04%	94.09%	90.52%	25,372	64,364	18.17%	86.59%	81.28%
MPSTAN w/o Mobility	5030	9845	7.09%	99.78%	99.65%	8147	14,895	11.17%	**99.56%**	99.16%
MPSTAN w/o MPG	4399	9033	6.71%	99.77%	99.70%	**7640**	**14,456**	**10.70%**	99.55%	**99.21%**
MPSTAN	**3960**	**8255**	**6.38%**	**99.80%**	**99.75%**	7711	14,463	10.73%	99.55%	99.20%
	T = 15 days					T = 20 days				
Model	MAE	RMSE	MAPE	PCC	CCC	MAE	RMSE	MAPE	PCC	CCC
MPSTAN w/o Phy-All	22,876	58,160	19.10%	88.68%	85.34%	27,659	60,632	24.83%	89.30%	83.16%
MPSTAN w/o Phy-Loss	18,526	32,033	20.19%	99.15%	95.58%	22,138	37,753	24.06%	97.54%	93.59%
MPSTAN w/o Phy-Model	27,509	63,056	21.84%	88.68%	80.58%	27,425	61,194	24.36%	88.96%	82.69%
MPSTAN w/o Mobility	11,054	20,240	15.33%	99.19%	98.39%	**11,859**	**22,477**	**18.37%**	98.71%	**98.02%**
MPSTAN w/o MPG	10,441	18,984	14.92%	**99.25%**	98.59%	13,064	23,702	18.98%	**98.87%**	97.75%
MPSTAN	**10,148**	**18,460**	**14.68%**	**99.25%**	**98.68%**	12728	22923	18.68%	98.81%	97.91%

**Table 5:** Ablation study on the Japanese dataset.

	The Japanese dataset									
	T = 5 days					T = 10 days				
Model	MAE	RMSE	MAPE	PCC	CCC	MAE	RMSE	MAPE	PCC	CCC
MPSTAN w/o Phy-All	3326	10,410	26.59%	92.27%	66.80%	3201	9632	29.52%	92.06%	68.65%
MPSTAN w/o Phy-Loss	**928**	**2024**	**15.81%**	96.05%	**95.90%**	**1196**	**2620**	**22.31%**	**93.70%**	**93.50%**
MPSTAN w/o Phy-Model	3674	11,714	28.11%	91.84%	62.93%	3896	10,762	45.96%	91.48%	64.93%
MPSTAN w/o Mobility	1142	**2309**	21.93%	**98.46%**	95.60%	**1273**	**2633**	27.24%	**96.77%**	**94.33%**
MPSTAN w/o MPG	1047	2339	19.18%	96.52%	95.44%	**1216**	**2630**	**23.49%**	**94.52%**	**93.90%**
MPSTAN	1016	2311	16.91%	96.74%	95.60%	1356	3016	24.34%	93.38%	92.27%
	T = 15 days					T = 20 days				
Model	MAE	RMSE	MAPE	PCC	CCC	MAE	RMSE	MAPE	PCC	CCC
MPSTAN w/o Phy-All	3435	9796	35.25%	91.37%	67.63%	3054	7736	41.09%	**90.29%**	74.42%
MPSTAN w/o Phy-Loss	1774	3941	32.28%	84.22%	83.44%	1928	4383	41.30%	**86.70%**	84.83%
MPSTAN w/o Phy-Model	3897	11,099	42.38%	90.35%	63.40%	4273	10,664	69.34%	**86.83%**	63.25%
MPSTAN w/o Mobility	**1100**	**2271**	**24.42%**	**96.25%**	**95.31%**	**1319**	**2958**	**26.72%**	**93.80%**	**92.42%**
MPSTAN w/o MPG	**1391**	3379	**25.48%**	**91.89%**	90.44%	**1786**	4073	**31.19%**	**87.89%**	**85.75%**
MPSTAN	1465	3104	28.29%	91.84%	91.29%	1854	4014	34.67%	85.78%	84.97%

**Table 6:** Comparison of model complexity at T = 5 days on the US dataset.

	Neural Network Parameters	Training Time Consumption
GRU	32 K	108 s
GraphWaveNet	270 K	122 s
STGODE	456 K	328 s
CovidGNN	119 K	20 s
ColaGNN	277 K	132 s
STAN	949 K	1560 s
PatchTST	6310 K	390 s
Crossformer	14,774 K	1404 s
MPSTAN	24 K	735 s

## Data Availability

Data are contained within the article.
